# Single-Particle Functionality Imaging of Antibody-Conjugated Nanoparticles in Complex Media

**DOI:** 10.1021/acsabm.2c00830

**Published:** 2023-01-03

**Authors:** Laura Woythe, Marrit M. E. Tholen, Bas J. H. M. Rosier, Lorenzo Albertazzi

**Affiliations:** † Department of Biomedical Engineering, Institute for Complex Molecular systems (ICMS), Eindhoven University of Technology, 5600 MB Eindhoven, Netherlands; ‡ Institute of Bioengineering of Catalonia (IBEC), The Barcelona Institute of Science and Technology (BIST), 08028 Barcelona, Spain

**Keywords:** active targeting, biomolecular corona, TIRF microscopy, cell selectivity, nanoparticle conjugation, heterogeneity

## Abstract

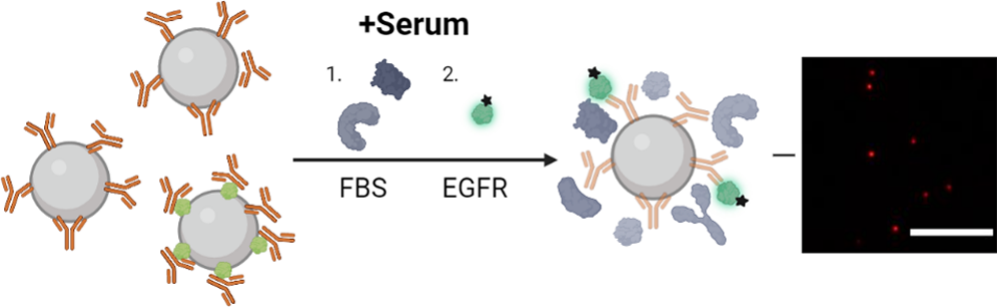

The properties of nanoparticles (NPs) can change upon contact with serum components, occluding the NP surface by forming a biomolecular corona. It is believed that targeted NPs can lose their functionality due to this biological coating, thus losing specificity and selectivity toward target cells and leading to poor therapeutic efficiency. A better understanding of how the biomolecular corona affects NP ligand functionality is needed to maintain NP targeting capabilities. However, techniques that can quantify the functionality of NPs at a single-particle level in a complex medium are limited and often laborious in sample preparation, measurement, and analysis. In this work, the influence of serum exposure on the functionality of antibody-functionalized NPs was quantified using a straightforward total internal reflection fluorescence (TIRF) microscopy method and evaluated in cell uptake studies. The single-particle resolution of TIRF reveals the interparticle functionality heterogeneity and the substantial differences between NPs conjugated with covalent and noncovalent methods. Notably, only NPs covalently conjugated with a relatively high amount of antibodies maintain their functionality to a certain extent and still showed cell specificity and selectivity toward high receptor density cells after incubation in full serum. The presented study emphasizes the importance of single-particle functional characterization of NPs in complex media, contributing to the understanding and design of targeted NPs that retain their cell specificity and selectivity in biologically relevant conditions.

## Introduction

Ligand-functionalized NPs can be used as drug delivery carriers to improve the selectivity toward target cells. However, after systemic administration, NPs properties can be influenced by their interaction with serum components (such as albumin, immunoglobulins, or apolipoproteins).^1,2^ This biological coating is referred to as biomolecular corona and provides NPs with a new physicochemical identity that influences the NP fate inside the body.^3^ One of the main concerns is that the biomolecular corona shields the functional ligand sites of targeted NPs, hindering the recognition of their target receptor. Understanding the ligand availability in serum is crucial for multivalent targeting strategies, where NP valency determines NP selectivity. Furthermore, the biomolecular corona is known to contain active biomolecules that have targeting properties themselves, potentially inducing undesired targeting effects.^4,5^ The unpredictable properties of the biomolecular corona formation and the obstruction of targeting ligands present one of the main bottlenecks that could affect the targeting efficacy of NPs in clinical studies.^6^ A better understanding of the influence of serum exposure on NP ligand functionality is required to improve their clinical success.

Methods that quantify the functionality of NPs in situ at a single-particle level are highly desired, as a significant heterogeneity exists between (1) the NP functionality after ligand conjugation^7,8^ and (2) the interaction of NP with serum components.^9^ Thus, average numbers are often not representative of the NP functionality of the entire batch and correlation with the uptake activity can be challenging if the molecular picture is not clearly understood.^10^

Single-molecule microscopy techniques, such as transmission electron microscopy (TEM) and single-molecule localization microscopy (SMLM), have been used to obtain detailed information about NP functional sites. The mapping of functional sites using TEM has been described using a gold NP probe that binds only to functional ligands on conjugated NPs.^11,12^ However, TEM microscopy is generally laborious and operates under non-native, dry conditions that could influence the integrity of NP ligands and is generally unsuited for NPs that cannot withstand these conditions, such as liposomes or micelles. Our group developed functional labeling approaches to quantify the ligand sites on NPs using different SMLM techniques.^8,13^ The functionality of antibodies conjugated to NPs was recently quantified using direct stochastic optical reconstruction microscopy (dSTORM) with nanometric resolution at a single-particle level, giving insights into NP heterogeneity beyond conventional ensemble characterization. Although offering a molecular resolution, these methods can be labor-intensive and require sophisticated sample preparation optimization, especially for imaging in complex biological media. Microscopy methods able to quantify NP properties at a single-particle level are a good compromise between throughput and resolution, providing broader accessibility of the technique while having a fundamental contribution to solving questions regarding NP functionalization^14^ and NP interactions with the biological environment.^15^

Here, we studied the influence of serum exposure on NP ligand functionality using single-particle total internal reflection (TIRF) microscopy and flow cytometry cell uptake. First, the effects of full serum exposure were systematically investigated by quantifying the functional characteristics of antibody-conjugated NPs on a single-particle level before and after serum incubation using a functional labeling approach. Specifically, only antibodies that were accessible and functional after serum exposure were labeled and imaged with minimal purification artifacts due to the absence of harsh centrifugation steps. In contrast to a recent report describing the loss of functionality of covalently attached antibodies to NPs,^16^ we found that antibodies preserve a certain percentage of their functionality on the NPs used in this study after serum incubation, with a strong dependence on the amount of antibody conjugated. The method is further explored to compare different antibody conjugation methods, namely, carbodiimide-based chemical conjugation, physical adsorption, and protein-G-mediated antibody attachment. We found that only antibodies that were covalently conjugated to NPs were able to withstand serum exposure and maintain their targeting capabilities to a certain extent. The targeting functionality is further reduced by spiking soluble EGFR fractions into serum that are known to be present in human circulation. Finally, cell uptake studies revealed that only NPs with a high concentration of covalently conjugated cetuximab present specificity and selectivity toward high EGFR density cells even after serum pre-incubation.

This study investigates the quantification of single-particle functionality under complex biological conditions, screening of different NP conjugation approaches, and the effects of serum incubation of targeted NPs on cell uptake. We present TIRF functional microscopy as a valuable and accessible tool that could potentially be used as a routine screening procedure to evaluate the targeting properties of NPs in complex media such as full serum. The gained single-particle insights can be used to design multivalent NPs that can withstand the effect of serum on their targeting properties, closing the gap between targeted NP development and clinical applications.

## Materials and Methods

### Materials and Reagents

Sicastar-greenF COOH (200 nm, 25 mg/ml) and Sicastar-greenF NH_2_ nanoparticles (200 nm, 25 mg/mL) were obtained from Micromod Partikeltechnologie GmbH. Cetuximab antibody (Erbitux, Merck) was kindly provided by Prof. Maarten Merkx (Eindhoven University of Technology). Tris(2-carboxylethyl)phosphine hydrochloride (TCEP), 1-ethyl-3-(3-dimethylaminopropyl)-carbodiimide (EDC), tris(hydroxymethyl)-amino-methane (Tris Base), bovine serum albumin (96% purity), phosphate-buffered saline (PBS) tablets, cysteamine, catalase from bovine liver, glucose oxidase, and 4-morpholineethanesulfonic acid (MES) were purchased from Sigma-Aldrich. Sodium bicarbonate and poly-l-lysine solution (0.01%) were purchased from Merck. Sodium chloride was purchased from Sanal. PD-10 columns were purchased at GE Healthcare. Zeba spin columns (0.5 mL, 7 kDa MWCO), Human EGFR protein with Fc tag (ACROBiosystems EGR-H5252), Human EGFR protein with His tag (ACROBiosystems EGR-H5222), Alexa Fluor 647 NHS ester, DMEM (high glucose, no phenol red), penicillin–streptomycin, fetal bovine serum (qualified), Trypsin-EDTA (0.5%), HEPES buffer (1 M), sulfosuccinimidyl 4-(*N-*maleimidomethyl) cyclohexane-1-carboxylate (Sulfo-SMCC), Human IgG isotype control and Nunc cell culture flasks were purchased from Thermo Fisher Scientific. MCF-7 cells were kindly provided by Prof. Jaap den Toonder (Eindhoven University of Technology). MDA-MB-468 cells were obtained from ATCC (HTB-132). Plain AffiniPure Goat Anti-mouse antibody was purchased from Jackson Immunoresearch.

### Fluorescent Labeling of EGFR Protein, Cetuximab, and FBS

Cetuximab was buffer exchanged prior to labeling to sodium bicarbonate (pH 8.4 0.1 M) using a Zeba desalting column following the manufacturer’s instructions. EGFR, FBS, and cetuximab were incubated with Alexa Fluor 647 (AF647) NHS ester at 1:6, 1:8, and 1:8 mol ratio protein/dye, respectively, for 2 h at 22 °C and 400 rpm in a ThermoMixer (Eppendorf). Labeled proteins were purified from free dye using 2 consecutive Zeba desalting columns rinsed with PBS buffer according to the manufacturer’s protocol. The labeled proteins were measured with a NanoDrop One (Thermo) to determine the degree of labeling, using PBS as a blank. For EGFR-AF647 and cetuximab-AF647, degrees of labeling of 2.9 and 5.5 were obtained, respectively. For FBS, a degree of labeling was not determined.

### Chemical Conjugation of Cetuximab to Silica NP

Cetuximab was conjugated to silica-COOH NPs in MES buffer (50 mM, pH 5) via EDC chemistry. Silica NPs were washed with MES buffer by centrifugation for 10 min at 16,000*g* to remove storage solution. Next, NPs were resuspended in MES buffer (50 mM, pH 5) containing 2 mM EDC and incubated for 15 min at 22 °C and 400 rpm in a ThermoMixer. After activation, NPs were sonicated for 5 min in a bath sonicator and cetuximab antibody was added at different concentrations (50, 100, and 1000 cetuximab/NP according to theoretical estimations) and incubated for 2 h at 22 °C and 400 rpm in a ThermoMixer. As a control formulation (0 cetuximab /NP) a human IgG isotype control antibody was used at a 1000 antibody/NP ratio. Unconjugated antibodies were purified by washing with 25 mM HEPES buffer and centrifuging thrice at 16,000*g* for 15 min. NPs were resuspended at a final concentration of 2 mg/mL in 25 mM HEPES buffer and stored at 4 °C.

### Adsorption of Cetuximab to Silica NP

Cetuximab was physically adsorbed to silica-NH_2_ NPs in MES buffer (50 mM, pH 5). Silica NPs were washed with MES buffer by centrifugation for 10 min at 16,000*g* to remove storage solution. Next, NPs were resuspended in MES buffer (50 mM, pH 5), sonicated for 5 min in a bath sonicator, and cetuximab antibody was added at 1000 cetuximab/NP according to theoretical estimations and incubated for 2 h at 22 °C and 400 rpm in a ThermoMixer. As a control formulation, a human IgG isotype control antibody was used at a 1000 antibody/NP ratio. Unconjugated antibodies were purified by washing with 25 mM HEPES buffer and centrifuging thrice at 16,000*g* for 15 min. NPs were resuspended at a final concentration of 2 mg/mL in 25 mM HEPES buffer and stored at 4 °C.

### Recombinant Protein Cloning, Expression, and Purification of Protein G

The recombinant protein cloning, expression, and purification of protein G were performed as originally described by Wouters et al.^17^ Protein G was encoded in a pET28a vector and synthesized by GenScript. The pG construct was based on a synthetic monomeric variant containing a C-terminal cysteine and a photoactive unnatural amino acid for covalent crosslinking to antibodies (Supporting Figure S13). For protein G expression, the expression plasmid was co-transformed with the pEVOL-pBpF plasmid (kind gift from Peter Schultz, Addgene plasmid no. 31190), which contains a tRNA–tRNA synthetase pair enabling incorporation of p-benzoylphenylalanine (pBzF), in chemically competent *Escherichia coli* BL21(DE3) cells (Novagen). Bacteria were cultured in 0.5 L 2xYT medium (2.5 g of NaCl, 5 g of Yeast extract, 8 g of Peptone in 0.5 L dH_2_O) supplemented with 25 μg/mL chloramphenicol and 50 μg/mL kanamycin. Upon reaching an OD600 of 0.5–0.6, expression was induced using 1 mM isopropyl β-d-1-thiogalactopyramoside (IPTG), 0.02 w/v % arabinose, and 1 mM pBzF. After overnight expression at 20 °C and 250 rpm, the cells were harvested by centrifugation at 10,000*g* for 10 min. The cells were then lysed using BugBuster protein extraction reagent (Novagen) and Benzonase endonuclease (Novagen) for 1 h and subsequently centrifuged at 16,000*g* for 20–40 min. The protein was purified using subsequent Ni-affinity chromatography (Novagen, His-bind resin) and Strep-Tactin XT (IBA) purification of the protein was performed according to the manufacturer’s instructions. To start with, the cleared lysate was applied to a nickel-charged column, washed with wash buffer (1× PBS, 370 mM NaCl, 10% (v/v) glycerol, 20 mM imidazole, pH 7.4), and eluted with elution buffer (1× PBS, 370 mM NaCl, 10% (v/v) glycerol, 250 mM imidazole, pH 7.4). Then, the eluate was applied to a Strep-Tactin column. The column was washed with wash buffer (100 mM Tris–HCl, 150 mM NaCl, 1 mM EDTA, pH 8.0), and the protein was eluted with wash buffer supplemented with 50 mM biotin. Proteins were aliquoted in 500 μL fractions and stored frozen at −80 °C until further use. Absorption at 280 nm (ND-1000, Thermo Scientific) was used to calculate protein concentration, assuming an extinction coefficient of 15,570 M^–1^ cm^–1^. Purity was assessed on 4–20% SDS-PAGE precast gels (Bio-Rad) under reducing conditions, stained with Coomassie Brilliant Blue G-250 (Bio-Rad) (figure not shown). The molecular weight was confirmed using liquid chromatography quadrupole time-of-flight mass spectrometry (Waters ACQUITY UPLC I-Class System coupled to a Xevo G2 Q-ToF) by injecting a 0.1 μL sample into an Agilent Polaris C18A reversed-phase column with a flow of 0.3 mL/min and a 15–60% acetonitrile gradient containing 0.1% formic acid.

### Protein G Functionalization of Silica-Amino NPs

For conjugation of protein G to the surface of amino-functionalized silica NPs, the protein was first reduced using TCEP. 5 mM TCEP was added to 1 mL of protein and this solution was incubated for 1 h at 25 °C, under moderate shaking. Thereafter, the protein was desalted using a PD-10 column and reaction buffer (100 mM sodium phosphate, 25 μM TCEP, pH 7) according to the manufacturer’s protocol and the concentration of the product was determined on the basis of absorption at 280 nm, assuming an extinction coefficient of 15,470 M^–1^ cm^–1^. Meanwhile, a solution of 1 mg 200 nm green fluorescent amino NPs (40 μL) was concentrated to 100 mg/mL in 1× PBS, pH 7.2 (10 μL), and 10 nmol of Sulfo-SMCC in DMSO (10 μL) was added. The reaction was incubated for 30 min at room temperature with slow rotation and tilt. Excess Sulfo-SMCC was removed by washing with 400 μL of reaction buffer (16,500*g*, 5 min). The NPs were resuspended in 100 μL of reaction buffer containing 100 μM protein G. After 2 h of incubation, the NPs were washed twice with 1× PBS (16,500*g*, 5 min). Finally, protein-G-conjugated NPs were reconstituted in PBS to reach a final concentration of 25 mg/mL and stored at 4 °C.

### Protein-G-Mediated Conjugation of Cetuximab to Silica NPs

Cetuximab was incubated with protein-G-conjugated NPs in MES buffer (50 mM, pH 5), sonicated for 5 min in a bath sonicator and cetuximab antibody was added at 1000 cetuximab/NP according to theoretical estimations and incubated for 2 h at 22 °C and 400 rpm in a ThermoMixer. As a control formulation, a human IgG isotype control antibody was used at a 1000 antibody/NP ratio. Unconjugated antibodies were purified by washing with 25 mM HEPES buffer and centrifuging thrice at 16,000*g* for 15 min. NPs were resuspended at a final concentration of 2 mg/mL in 25 mM HEPES buffer and stored at 4 °C.

### Serum Incubation of NPs

Silica-cetuximab NPs were incubated in 100% fetal bovine serum at a concentration of 100 μg/mL NPs for 2 h and 37 °C to induce the formation of a biomolecular corona.

### Incubation of Silica-Cetuximab NPs with EGFR-AF647 Probe

Silica-cetuximab NPs were incubated with EGFR-AF647 probes to determine their functionality in the absence and presence of serum pre-incubation. Silica-cetuximab NPs with and without serum pre-incubation were first sonicated in a bath sonicator for 5 min. Silica-cetuximab NPs without serum pre-incubation were incubated with 16 pmol of EGFR-AF647 and 0.5% bovine serum albumin to block unspecific interactions for 1 h at 25 °C and 400 rpm in a ThermoMixer. Silica-cetuximab NPs with serum pre-incubation were incubated with 16 pmol of EGFR-AF647 for 1 h at 25 °C and 400 rpm in a ThermoMixer. NPs were sonicated in a bath sonicator for 5 min to aid redispersion and imaged the same day.

### TIRF Imaging

Coverslips (22 mm × 22 mm, #1.5) were cleaned by sonication in isopropanol for 20 min and dried under nitrogen flow, and microscope slides (76 mm × 26 mm, thickness 1 mm) were cleaned using an isopropanol-soaked tissue. An imaging chamber was prepared by attaching one coverslip to a microscope slide using double-sided scotch tape. This created a chamber of approximately 20 μL volume. The microscopy slide was then filled with 0.01% solution of poly-l-lysine to coat the coverslips for better NP attachment for 10 min at room temperature. The imaging chamber was then washed with HEPES buffer and silica-cetuximab NPs incubated in serum and labeled with EFGR probe were added and allowed to attach for 20 min. For silica-cetuximab NPs without serum pre-incubation, the microscopy chamber was first incubated with poly-l-lysine, then with FBS for 10 min, and finally, NPs were added. Nonattached NPs were washed away by flushing HEPES buffer through the microscopy chamber and the sample was imaged. Imaging was performed with a Nikon N-STORM system configured for TIRF imaging and equipped with a perfect focus system. The TIRF angle was adjusted to maximize the signal-to-noise ratio. A Nikon 100×, 1.4 NA oil immersion objective was employed to collect the fluorescence signal, which was passed through a quad-band pass dichroic filter (97,335, Nikon) and recorded on an Andor EMCCD camera (ixon3) with pixel size 160 nm and a region of interest of 256 × 256 pixels or 512 × 512 pixels. TIRF images of 488 (silica NPs) and 647 (EGFR-AF647 or cetuximab-AF647) were acquired at 2% laser power and 100 ms exposure of different fields of view.

### Single-Particle Image Analysis

TIRF images from the 488 and 647 channels were imported into ImageJ and analyzed using the ComDet plugin v.0.5.5, an open-source ImageJ plugin for the colocalization analysis of fluorescent spots of microscopy images.^18^ The plugin was used to (1) detect the NP position using the 488 channel, (2) colocalize the EGFR-AF647 signal with the NP position, and (3) extract the integrated intensity of the EGFR-AF647 spots. The intensity threshold for NP detection was set to 5 times the background SD with an approximate particle size of 2 pixels. The integrated intensity was calculated by the software as the sum of all pixel intensity insight the thresholded area minus the average background, calculated from the average intensity of pixels along the detected NP region of interest. The results table was exported and analyzed using Origin 2020 software. First, integrated intensities with a negative value were set to intensity 0. These negative values can result from an uneven background signal detection, for example from fluorescent probe binding to the surface. Next, the integrated intensities of the EGFR-647 signal in the presence of serum were multiplied by a correction factor to account for the different signal intensities of EGFR-AF647 molecules in HEPES buffer compared to full serum medium. The correction factor was calculated by measuring the EGFR-AF647 intensity diluted in HEPES/BSA buffer or full serum buffer in a plate reader to simulate the TIRF imaging conditions (Supporting Figure S14). From the fluorescence peak intensity, it was determined that the intensity of EGFR-AF647 was 17.4% higher in full serum than in HEPES/BSA buffer; thus, the correction factor was set at 0.174. Next, the fluorescence intensity was normalized by the average of the 15 highest intensity values as previously described elsewhere.^19^ To verify this, the raw data was plotted (Supporting Figure S5). For comparison, the mean values of each group were used. To be able to assess the statistical significance of the results, the log normal distributions were transformed to the logarithmic and a paired t-test was performed (Supporting Figure S6 and Table S1).

### NP Uptake by Flow Cytometry

MCF-7 and MDA-MB-468 cells were cultured in DMEM (high glucose, no phenol red) supplemented with 10% FBS and penicillin–streptomycin (100 U/mL) at 37 °C and 5% CO_2_. For NP uptake experiments, MCF-7 and MDA-MB-468 cells were seeded in a 48-well plate at a density of 45,000 cells/well and incubated for 48h at 37 °C and 5% CO_2_. The cells were washed once with PBS and incubated with NPs with and without pre-incubation in 100% serum, diluted to a final concentration of 50 μg/mL NPs/well in DMEM without FBS (final volume 100 μL/well), for 90 min at 37 °C and 5% CO_2_. For comparison, NP uptake of nonserum pre-incubated NPs was additionally performed in the presence of 0.5% BSA (Supporting Figure S12). After NP incubation, the cells were washed with PBS, detached using trypsin, and centrifuged for 5 min at 300*g*. The cells were resuspended in 300 μL of BSA 1% in PBS and kept on ice before the flow cytometry measurement. For each condition, a minimum of 20,000 cells were measured on a BD FACSCanto II configured for FITC detection. Flow cytometry data were analyzed using FlowJo (version 10.7.1).

## Results

**Figure 1:**
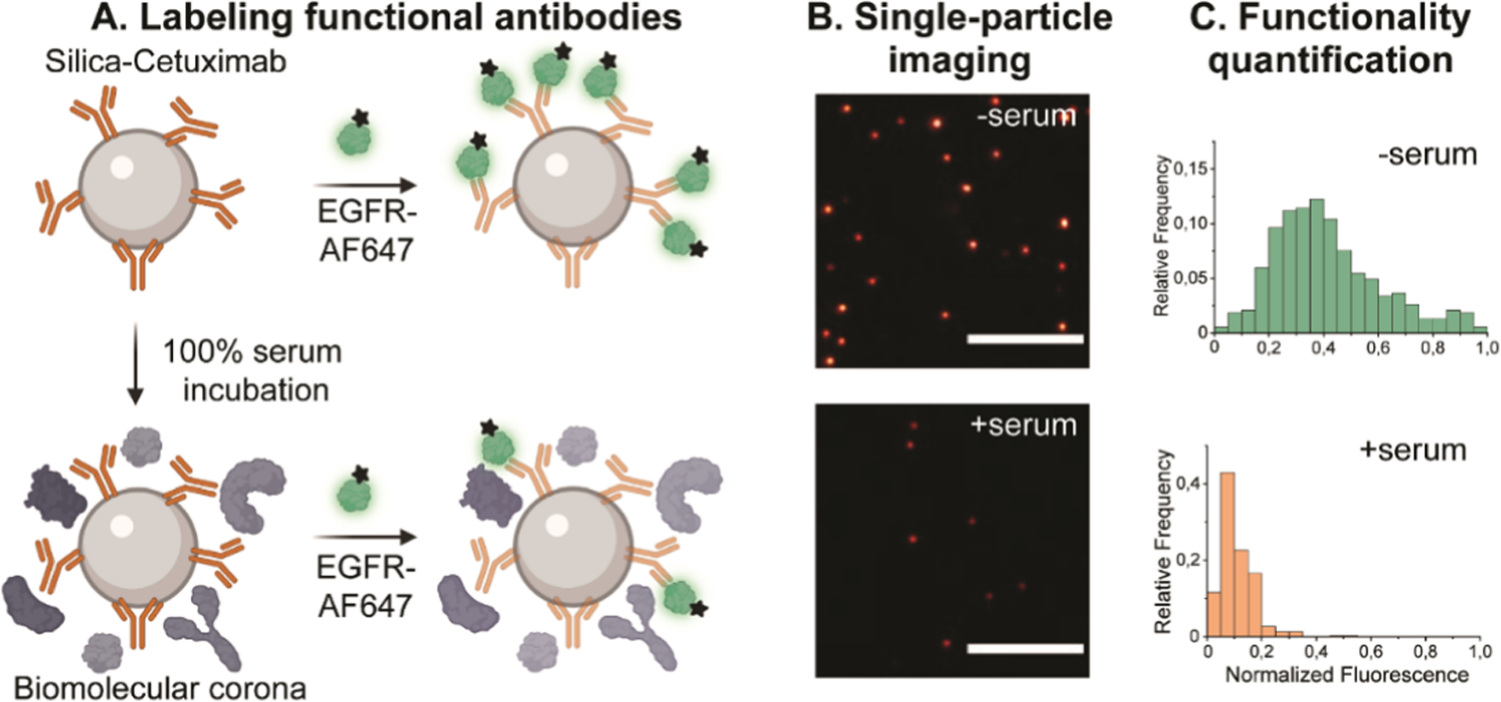
NP functionality imaging and quantification in complex medium. (A) Schematic representation of functional labeling of silica-cetuximab NPs with and without protein corona formation. To visualize accessible cetuximab antibodies, a fluorescent EGFR probe was used for detection. (B) Individual NP functionality was imaged using TIRF microscopy (scale bar, 10 μm) and (C) quantified at the single-particle level to determine the functionality before and after exposure to serum and biomolecular corona formation.

To understand NP targeting in the biological environment, it is essential to know the influence of serum components on the NP ligand functionality. The formation of a biomolecular corona around ligand-conjugated NPs can shield their functionality, hindering their target recognition (i.e., cell surface receptors). Previously, we developed a functional labeling protocol to image the functional sites of cetuximab-conjugated NPs.^8^ Here, this protocol was extended to image the silica-cetuximab functional sites after serum exposure, providing better insights into the NP targeting ability in complex biological environments. A schematic representation of the assay is presented in Figure 1. First, silica-cetuximab NPs were incubated either in buffer (no biomolecular corona condition) or 100% serum at 37 °C for 2 h, which was enough time for a biomolecular corona to form, as previously described.^9^ Next, the NP functionality was imaged using a fluorescent EGFR probe consisting of a recombinant extracellular part of the receptor recognized by the cetuximab fragment antigen binding (Fab) regions (Figure 1A). Thus, only accessible and functional cetuximab Fab sites were labeled, while unfunctional or shielded cetuximab by serum components remained undetected. NPs were visualized at the single-particle level using total internal reflection fluorescence (TIRF) microscopy (Figure 1B), allowing the quantification of functional cetuximab antibodies before and after serum incubation, providing an indication of the percentage of NP functionality loss due to biomolecular corona shielding (Figure 1C). Notably, our method does not require any harsh NP purification steps, thus the functionality is assessed in the native state of the formed biomolecular corona after serum exposure.

**Figure 2:**
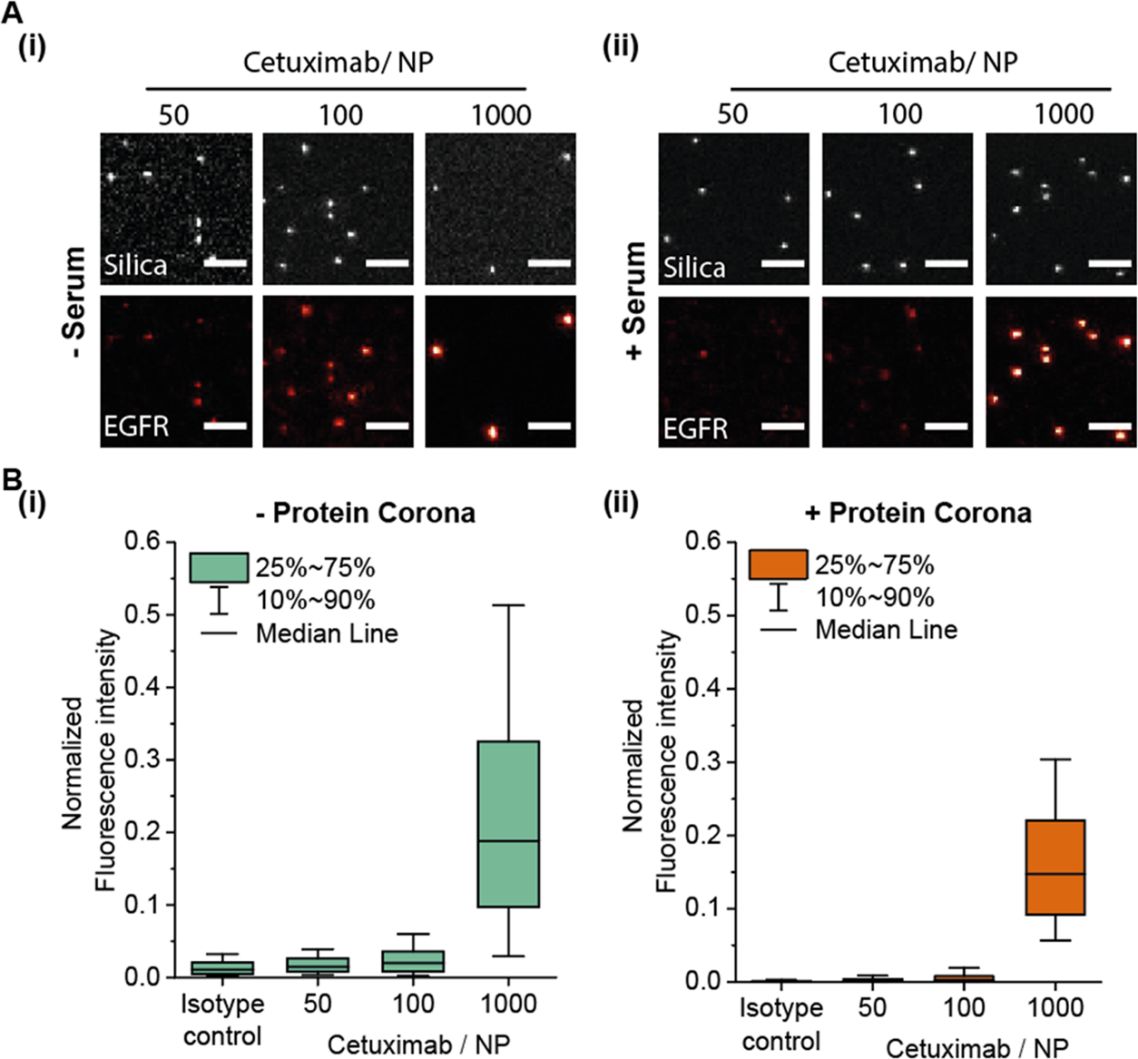
Influence of cetuximab concentration on NP functionality in the absence and presence of serum incubation. Cetuximab antibodies were conjugated to NPs via an EDC-based covalent chemical reaction. (A) Representative TIRF images of silica NPs incubated (i) without and (ii) with serum and detected with a fluorescent EGFR-AF647 probe. To identify the NP position, fluorescent silica NPs were used. Scale bar, 5 μm. (B) Box plots indicating normalized fluorescence intensity of labeled EGFR probe per NP formulation (i) without and (ii) with serum incubation. As a control, NPs functionalized with a human isotype control antibody (1000 antibodies/NP, estimated theoretically) were used. Box represents 25 to 75 percentile and whiskers 10 to 90 percentile. Box line indicates median normalized fluorescence intensity. A minimum of 300 NPs were measured for each condition in different fields of view. Note that *x*-axis is not to scale but represents individual conditions.

First, the concentration of cetuximab conjugated to NPs was varied to investigate the difference between a low and high concentration of cetuximab on the functionality loss after full serum exposure (Figure 2). Therefore, cetuximab was conjugated to silica NPs (200 nm diameter, carboxylic acid functionalized) at different amounts using a carbodiimide-based chemical reaction, specifically 1-ethyl-3-(3-dimethylaminopropyl)-carbodiimide (EDC) chemistry. This resulted in random orientations of cetuximab on the NP surface, as the EDC-activated NPs react stochastically with any of the available lysine residues present in the antibody. The resulting silica-cetuximab NPs were either incubated with buffer (− serum) or full serum (+ serum) at 37 °C for 2 h, which enabled the biomolecular corona formation. Subsequently, the labeled EGFR-Alexa Fluor 647 (EGFR-AF647) probe was added to the NP formulations to visualize the functional cetuximab sites. Silica-cetuximab NPs were added to a microscopy chamber and imaged by TIRF microscopy without any further purification steps. Functional NPs were detected by the spatial colocalization of fluorescent silica NPs cores (FITC) with the EGFR probe fluorescence (AF647). One limitation that needs to be taken into account is the possible bias of nanoparticles sticking to the glass. Due to the addition of serum, some particles might be repelled from the glass surface. A representative TIRF image of single NP labeled with EGFR-AF647 probe is shown in Figure 2A, where each fluorescent dot represents one NP. The fluorescence intensity originating from silica cores is presented in greyscale, while EGFR-AF647 is shown in red. In the absence of serum, EGFR-AF647 signal co-localized with the NP position (Figure 2A(i)). However, after serum incubation, the EGFR-AF647 signal was strongly reduced at low cetuximab concentration, indicating that NPs most likely lost their functionality due to the shielding of serum biomolecules (Figure 2A(ii)). On the other hand, at a high cetuximab concentration (1000 cetuximab/ NP), there was a clearly observable EGFR-AF647 signal, indicating that the functionality was partially maintained. All groups are statistically different from each other and between with and without serum.

The EGFR-AF647 intensity was then quantified to extract single-particle information on the NP functionality (Figure 2B). Additionally, a control formulation was measured consisting of bare NPs and NPs conjugated with a human isotype control antibody (Figure 2B and Supporting Figures S1 and S2). The reproducibility of the TIRF functionality assay and quantification workflow was confirmed for NPs with 1000 cetuximab/ NP (Supporting Figure S3). Furthermore, the amount of serum that bound to the particles was quantified using fluorescently labeled FBS (Supporting Figure S8), which resulted in comparable amounts of FBS bound to the particles. As expected, the antibody functionality was reduced after the NP exposure to serum in all tested conditions (Figure 2B(ii)). In the highest cetuximab concentration (1000 cetuximab/ NP), an average mean functionality loss of 30.5% compared to no serum exposure was observed. It is important to note that the distribution is changed from a Gaussian to a log normal distribution and the tail of the distribution is reduced, as is represented in Supporting Figure S6, which can be explained by an increase in heterogeneity of the sample. Thus, it is expected that the cell targeting capabilities after serum exposure will be considerably hindered. More strikingly, the functionality was almost completely lost at low cetuximab concentrations (50 and 100 cetuximab/ NP) after serum incubation (a zoom into the *y*-axis is displayed in Supporting Figure S4). The control formulation using the bare particles and an isotype control antibody showed minor EGFR-AF647 unspecific probe binding in buffer, while unspecific binding was fully blocked after serum incubation (Supporting Figures S1 and S2). Compared between isotype control and bare particles, the nonspecific binding is higher for the bare particles, with a difference in means of 20.8% for the serum-free condition and 700% for the serum condition. This is explained by the higher nonspecific interaction of bare particles caused by the accessible silica.

The results in this section showed that using too few targeting ligands on the NP surface can result in reduced or no targeting capabilities of the NP formulation after exposure to serum components. On the other hand, using a higher concentration of ligands the functionality was reduced, but partially maintained. Approximately 70% of the NP cetuximab still preserved their functionality and could recognize the target receptor even after incubation in full serum.

Next, we compared the functionality of nonoriented (i.e., EDC-mediated) against oriented silica-cetuximab conjugation approaches. It is expected that the NP functionality will differ considerably depending on the conjugation strategy, with oriented methods providing more control over maximizing the ligand functionality on the NP surface. For example, adaptor molecules such as protein A or protein G can be used to interact with a defined antibody region, leaving the receptor-recognition sites exposed and accessible on the NP surface. Furthermore, little information is known about the NP serum interaction of formulations prepared using different conjugation chemistries.

To investigate the effect of antibody orientation on NP functionality, we conjugated cetuximab to silica NPs using either EDC chemical conjugation, physical adsorption to amino-functionalized NPs, or molecular recognition by protein-G-functionalized NPs. While EDC-based conjugations and physical adsorption lead to the stochastic orientation of cetuximab on the NP surface, protein G binds to the fragment crystallizable (Fc) region of cetuximab, mediating a favorable orientation for many Fab sites to be functional. Based on the previous results, we used the highest cetuximab concentration in our studied range (1000 cetuximab/ NP) for NP conjugation via different methods. As a control condition, human isotype antibody was conjugated using the same methods and showed minimal binding of the EGFR-AF647 probe (Supporting Figure S9). Furthermore, the amine-functionalized bare particles were analyzed (Supporting Figure S7), which showed minor binding of the probe before incubation with serum and negligible binding after serum incubation. However, again, compared to the isotype control, the mean fluorescent intensity is higher for the bare particles.

**Figure 3:**
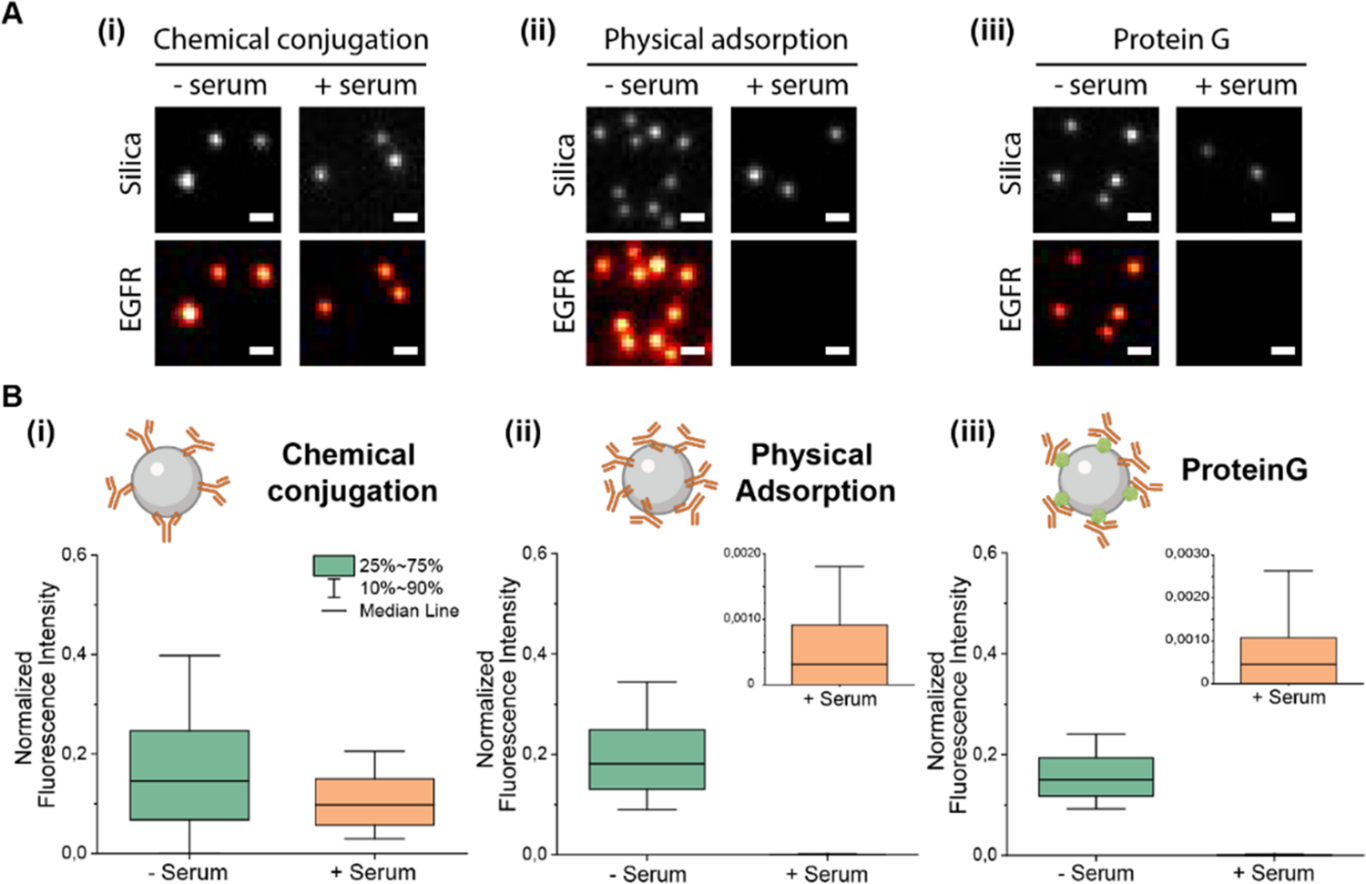
Evaluation of NP functionality using different conjugation approaches before and after exposure to serum. (A) Representative TIRF images of NP functionality after (i) chemical conjugation, reproduced in a similar way as for Figure 2, (ii) physical adsorption, or (iii) protein-G-mediated conjugation. Fluorescence silica NPs were used as a reference for the NP position. Scale bar, 1 μm. (B) Box plots indicating normalized fluorescence intensity of labeled EGFR probe with and without serum incubation using (i) chemical conjugation, (ii) physical adsorption, or (iii) protein-G-mediated cetuximab attachment. Box represents 25–75 percentile and whiskers 10–90 percentile. Box line indicates median normalized fluorescence intensity. A minimum of 170 NPs were measured for each condition in different fields of view.

In the absence of serum, we observed that NP functionality was similar between the three conjugation approaches (Figure 3), although the protein G particles display a more narrow distribution. This can be explained by a more homogeneous antibody binding, albeit with fewer antibodies. We observed a slightly higher median functionality in NP conjugated via physical adsorption compared to the other approaches. However, this slight difference might originate from a different number of total cetuximab conjugated to the NPs surface rather than an increased functionality, which is quantified with this assay. Conjugating NPs using directly labeled cetuximab (cetuximab-AF647) revealed less cetuximab conjugated per NP via the protein-G-mediated approach (Supporting Figure S10).

Interestingly, after exposure to full serum, cetuximab adsorbed physically or via protein G to NPs displayed almost no functionality (Figure 3(ii,iii)). To elucidate whether the functional cetuximab sites were blocked or the full antibodies were displaced from the NP surface by other serum proteins, NPs were conjugated with cetuximab-AF647 using the different conjugation methods and exposed to full serum. These results revealed that physically adsorbed and protein G adsorbed cetuximab were absent from the NP surface after incubation in serum (Supporting Figure S10).

The complex composition of serum often includes soluble receptor fractions, whose effects are relatively unexplored in targeted drug delivery. It is known that some receptors, such as EGFR, are expressed by cells as a soluble version of their extracellular domain that is shed into the bloodstream and capable to interact with their ligands.^20^ Additionally, EGFR shedding is also observed as a result of the cleavage of its membrane receptor. Although their mechanism and biological role are not clearly understood, this could result in a potential bottleneck for current targeted therapies since they maintain a high affinity for their corresponding ligands (i.e., EGF or cetuximab). Soluble EGFR (sEGFR) can potentially interact with ligand-functionalized NPs, resulting in the blocking of the NP targeting functionality and thus a reduced selective cell recognition. Concentrations of between 21.3 and 94.1 ng/mL have been measured in primary breast cancer patients.^21^

**Figure 4:**
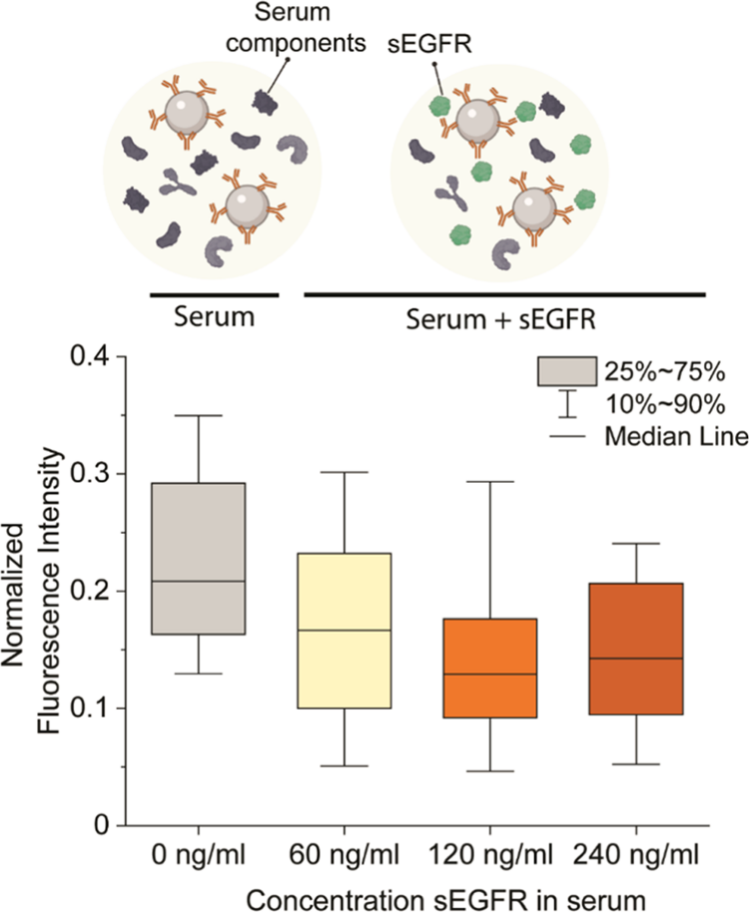
Box plots showing NP functionality of silica-cetuximab after exposure to serum containing different concentrations of soluble EGFR. Silica-cetuximab NPs were chemically bound at a concentration of 1000 cetuximab/ NP. Box represents 25% to 75% percentile and whiskers 10% to 90%. Box line represents median normalized fluorescence intensity. A minimum of 100 NPs were measured for each condition in different fields of view.

To this end, we studied the effect of the concentration of free sEGFR spiked into serum on NP ligand functionality (Figure 4). First, the conditioned serum was then incubated with NPs functionalized with 1000 cetuximab/ NP via chemical conjugation. Subsequently, the remaining active sites were labeled with the fluorescent EGFR-AF647 probe. We saw that in the lowest sEGFR concentration studied (60 ng/mL), the median functionality was reduced up to 20% on average with respect to incubation with serum without sEGFR (Figure 4). From the distribution, it can be observed that the whole population is reduced in signal. Furthermore, the maximum effect was seen at 120 ng/mL, where 39% of the median functionality was lost. These differences might originate from a skewed distribution with a tail of super-functional particles. From these distributions, it seems that these particles are targeted more, which is shown in the reduction in the intensities in the tail at 120 ng/mL, which could be due to a different kinetic interaction of these particles compared to particles with lower functionality.

Finally, we evaluated the NP bioaccumulation in two distinct cell lines using flow cytometry, namely, the highly expressing EGFR cells MDA-MB-468 and the low-expressing EGFR cells MCF-7. Following the previous protocol, NPs were pre-incubated in serum and subsequently diluted in cell culture medium before addition to the cells, without intermediate purification steps that could bias the results. For comparison, we use low (50 ab/ NP) and high (1000 ab/NP) concentration functionalization using EDC-based chemical conjugation and high (1000 ab/NP) concentration of physically adsorbed antibodies.

**Figure 5:**
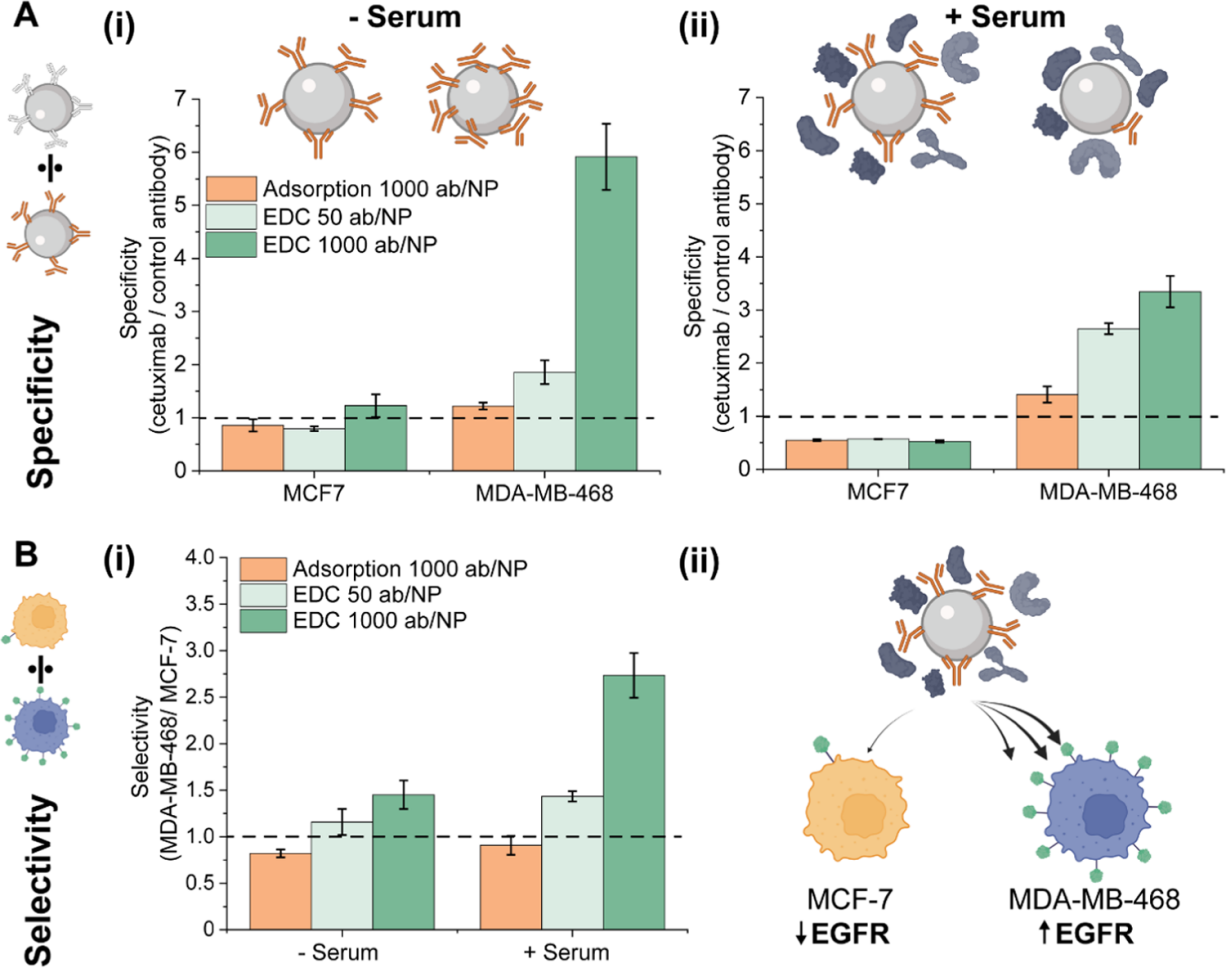
Specificity and selectivity of NP bioaccumulation with and without pre-incubation with full serum. NPs were functionalized using 1-ethyl-3-(3-dimethylaminopropyl)-carbodiimide (EDC)-based chemical conjugation at low (50 antibodies/NP) or high (1000 antibodies/NP) or physical adsorption (1000 antibodies/NP). (A) Bioaccumulation specificity in MCF-7 and MDA-MB-468 cells (i) without and (ii) with serum pre-incubation of NPs. Specificity was defined as fold increase bioaccumulation of silica-cetuximab NPs compared to control antibody NPs. (B) Selectivity of NPs toward MDA-MB-468 cells. Selectivity was defined as a fold increase in bioaccumulation of NPs in MDA-MB-468 compared to MCF-7 cells. (i) Bioaccumulation of different NP formulations was compared without and with serum pre-incubation. (ii) Schematic illustration of increased bioaccumulation in high EGFR expressing MDA-MD-468 cells compared to low EGFR expressing MCF-7 cells after NP serum pre-incubation.

NP cell bioaccumulation was evaluated by measuring the fluorescence intensity of labeled silica NPs per cell, with the signal intensity proportional to the internalized number of NPs. We defined two targeting parameters to understand NP targeting after serum exposure: NP specificity ratio and cell selectivity ratio. The specificity ratio determines the degree of nonspecific interactions between the NP and the cells and was defined as the fluorescence intensity fold increase of a silica-cetuximab NP with respect to a nontargeted particle functionalized with an isotype control antibody (Figure 5A). The selectivity ratio is indicative of cell preference for NP internalization and was defined as the fluorescence intensity fold increase of NP uptake in high EGFR expressing cells (MDA-MB-468) compared to control cells with minimal EGFR expression (MCF-7) (Figure 5B).

Specific targeting results from the increased NP bioaccumulation through the cetuximab-EGFR interaction. Without serum pre-incubation, we observed that MDA-MB-468 targeting using chemically conjugated silica-cetuximab NPs was 2 to 6-fold higher compared to the control antibody NPs (Figure 5A(i)). Surprisingly, NPs with adsorbed cetuximab did not lead to increased specificity in the absence of serum incubation. The results showed that NP bioaccumulation was equally high for targeting (silica-cetuximab) and nontargeting control NPs (Figure 5 and Supporting Figure S11), which could indicate that adsorbed cetuximab antibodies were present (Figure 3b(ii)) but the bioaccumulation was mainly driven by non-EGFR mediated interactions. As expected, targeting was unspecific in MCF-7 cells for all NP formulations due to the absence of EGFR receptors on these cells. The highest unspecific bioaccumulation was observed in adsorbed cetuximab and control antibodies, following a similar trend as observed in MDA-MB-468 cells.

In the case of pre-incubation with serum, specificity in MDA-MB-468 cells increases slightly for low concentrations but decreases visibly for high concentrations of chemically conjugated cetuximab (Figure 5A(ii)). This resulted from the decreased overall cell bioaccumulation of silica-cetuximab and control NPs, which could be attributed to the formation of a biomolecular corona increasing the antifouling properties of NPs (Supporting Figure S11). No specific targeting was observed for NPs with physically adsorbed antibodies, as expected from the absence of antibodies on the NPs after serum incubation (Figure 3b(ii)). For MCF-7 cells, targeting after serum incubation of NPs remains unspecific, indicating that no off-targeting effects are occurring toward these cells due to the serum protein components. The reduction in specificity that is observed could be explained by additional receptor blocking in the serum conditions, or by starvation of the cells in the serum-free case.

Selective targeting is the ability of an NP formulation to discriminate between two cells. Thus, we aimed for increased bioaccumulation in MDA-MB-468 while reducing the bioaccumulation in MCF-7 cells to a minimum. Selectivity of NPs toward MDA-MB-468 cells without serum pre-incubation yielded merely 1.5-fold higher bioaccumulation compared to MCF-7 in NPs with a high concentration of chemically conjugated antibodies (Figure 5B(i)). When cetuximab was conjugated at a lower concentration or physically adsorbed, no selectivity toward MDA-MB-468 cells is observed. This observation is a consequence of the high unspecific bioaccumulation of NPs in MCF-7 cells (silica-cetuximab and control NPs) in the absence of serum (Supporting Figure S11).

After serum pre-incubation of NPs, the selectivity of silica-cetuximab NPs toward MDA-MB-468 cells was slightly increased. This effect can be attributed to blocking unspecific interactions of silica-cetuximab NPs with MCF-7 cells, while the specific interactions with MDA-MB-468 cells were maintained. At low cetuximab chemical conjugation, there was a balance between lower unspecific bioaccumulation in MCF-7 and the blocking of specific bioaccumulation in MDA-MB-468 due to serum protein shielding of functional cetuximab. This translated into only a minor fold increase in bioaccumulation. At higher cetuximab chemical conjugation, there were more specific cetuximab-EGFR interactions in MDA-MB-468 cells while unspecific bioaccumulation in MCF-7 cells remains in a similar range, thus selectivity toward MDA-MB-468 cells rises accordingly. For adsorbed silica-cetuximab NPs, there was no selectivity toward MDA-MB-468 cells due to the absence of adsorbed cetuximab after serum exposure, as discussed earlier.

## Discussion

The functional ligand density of NPs is an important parameter that should be considered in the biological environment. However, what fraction of the targeting ligands is still functional after exposure to the biological environment is often overlooked. Understanding the influence of a biomolecular corona formation on NP functionality is crucial to engineer targeted nanomedicines since losing the NP specificity and selectivity toward target cells can hinder their clinical translation. Here, antibody-conjugated NPs were exposed to full serum and tested for ligand functionality at a single-particle level and cell bioaccumulation.

First, the antibody amount conjugated to NPs was varied to investigate the functionality of the formulations before and after serum exposure. We found that only NPs with a high antibody conjugation retain to some extent their functionality. Previous reports suggest that a few ligands are better than too many.^22^ Too many ligands per NP can result in steric hindrance between ligands, off-targeting interactions, or increased recognition and uptake by the immune system. However, the functionality after serum exposure needs to be considered to optimize the ligand amount.

Next, covalent and noncovalent attachment methods were compared to determine their suitability for cell targeting under serum conditions. In particular, carbodiimide-based chemical conjugation, physical adsorption, and protein-G-mediated binding were evaluated for NP functionalization. It is shown that the physical adsorption of cetuximab to amino-functionalized or protein-G-functionalized silica NPs was not strong enough to withstand exposure to serum proteins. Thus, covalent binding of targeting ligands to the investigated silica NPs is necessary to preserve NP targeting functionality. One hypothesis is that the strong dynamic interaction of the serum biomolecules caused a displacement of the noncovalently conjugated cetuximab antibodies. As earlier reported, the replacement of proteins on the NP surface in favor of stronger adhering ones is a commonly observed effect during the biomolecular corona formation, referred to as the Vroman effect.^23^

Physical adsorption is a relatively easy and thus attractive approach to conjugate the NP surface. In contrast to our results, previous studies have shown excellent targeting properties of adsorbed antibodies. For example, Tonigold et al. demonstrate that pre-adsorbed antibodies on polystyrene NPs are less affected by serum biomolecule shielding than chemically conjugated antibodies.^16^ However, the NP material, in this case, could strongly affect adsorption phenomena. Hydrophobic materials such as polystyrene tend to promote strong noncovalent binding via hydrophobic interactions with proteins.^24^ Additionally, to improve the functionality of protein-G-conjugated antibodies in serum, small photo-cross-linkable protein G molecules that covalently bind with antibodies could be used to chemically link the antibodies on the NP surface while maintaining a high ratio of functional antibodies.^25^

Further, the functionality of NPs was reduced when incubated with serum containing soluble EGFR known to be present in circulation. This strongly indicates that the composition of the serum is an essential factor to consider in the NP targeting design. The role of soluble receptor variants is often overlooked in NP targeting. This observation emphasizes the need for using a higher targeting ligand number to enhance the targeting efficiency in an in vivo situation and avoid ligand blocking by serum components.

Finally, NP targeting was evaluated with and without a preformed biomolecular corona. Previously, it was observed that the biomolecular corona reduces unspecific interactions of nonfunctionalized NPs with the cell membrane.^26^ Here, we observed that serum molecules shield unspecific as well as specific interactions of targeted NPs, balancing out the increased specificity expected for targeted NPs. Thus, evaluating the interactions of NPs exposed to serum with cells is relevant to understanding the specific and unspecific targeting properties. Notably, the presented results were specific for the cell types and the serum used. Care needs to be taken in generalizing the observed effects, and every NP formulation should be evaluated for unspecific binding in the studied cell lines. Previous studies demonstrate that serum composition is highly patient-dependent and can be influenced by diet, sex, and medical conditions.^27−29^ Thus, the specific serum composition could drive off-targeting effects of serum-coated NPs.

Furthermore, a recent overview of ongoing biomolecular corona research pointed out that (1) research on NPs > 100 nm is scarce, (2) there is a lack of diversity of NP systems studied, with gold NPs being the most prominent studied system, and (3) single-protein studies dominate the research field.^24^ The added complexity of studying a wide range of NP system in biologically relevant media needs to be addressed by the development of new techniques able to quantify the consequences of biomolecular formation in biologically relevant scenarios. Here, we address some of the shortcomings of the current literature by developing a protocol that is compatible with many NP systems and with full serum incubation to study NP functionality at a single-particle level in complex biological media. Unlike single-component biomolecular studies, we found that biologically relevant media such as serum should be the main focus of NP targeting research. Interestingly, we observed that there is a dramatic increase of NP bioaccumulation in MCF-7 cells when NPs were pre-incubated with only bovine serum albumin (BSA) instead of full serum (Supporting Figure S12), which would by itself lead to false conclusions regarding NP bioaccumulation. Single-component biomolecular coronas are an easy tool to study the effect of each biomolecule in the body on NP targeting, but fail to represent the complexity such as interactions between molecules and dynamic changes in time.

At the same time, NPs can also be engineered only to attract a specific biomolecule composition that minimizes unspecific cellular interactions.^30^ However, the modulation of NP fate based on biomolecular corona composition is still challenging and largely under investigation. Especially the effect of serum components on targeted NP is of main interest due to the potential loss of functionality and the off-targeting effects of serum components that have intrinsic targeting properties to cell receptors (i.e., transferrin).

Here, we developed a single-particle functionality method to investigate the functionality loss of cetuximab-conjugated NPs after serum incubation. This understanding is essential in the design of multivalent NP targeting, which is based on the concept that multiple NP ligands are available to interact with multiple cell receptors to increase NP avidity and selectivity toward cells. We highlight that by tuning the ligand number and conjugation method, NP ligands can either lose or keep part of their functionality; thus, multivalent NP targeting can still be facilitated under certain conditions, even in complex media.

## Conclusions

This study shows that the functionality and targeting properties of cetuximab-functionalized NPs are considerably affected after full serum incubation. First, the functionality loss was investigated using single-particle TIRF microscopy and a functional labeling approach. For covalently attached cetuximab to NPs, the functionality was substantially lost after serum incubation at low antibody concentration (below 100 cetuximab/NP). However, it could be maintained up to 70% on average when antibodies were conjugated at a higher concentration to NPs (1000 cetuximab/ NP). In contrast, noncovalently attached cetuximab to silica NPs resulted in antibody detachment from the NP surface after serum incubation; thus, no remaining functionality was detected. Other factors such as soluble EGFR fractions in serum also decrease NP functionality due to their specific binding with cetuximab on the NP surface. Finally, cell uptake studies in EGFR low- and high-expressing cells confirmed that NPs with a high concentration of cetuximab retained cell specificity and selectivity toward high EGFR density cells after exposure to full serum. Here, the NP functional properties at a single-particle level were investigated using TIRF microscopy and confirmed in cell bioaccumulation studies, yielding insight into how the formation of a biomolecular corona can dictate the NP targeting toward cells of interest.

## Supporting Information Available

Isotype control, bare particle control, reproducibility, zoom-in of *y*-axis, raw data plots, curve fits, statistical analysis, fluorescent FBS, fluorescent cetuximab, cell experiment fluorescent intensity increase, amino acid sequence of protein G, and comparison fluorescence in buffers (PDF)
