# Supplementary material for: Single-Particle Functionality Imaging of Antibody-Conjugated Nanoparticles in Complex Media
Source: ACS Appl Bio Mater. 2023 Jan 3;6(1):171–81. doi: 10.1021/acsabm.2c00830 (PMC9846673; doi:10.1021/acsabm.2c00830)
Supplement: Supplementary file 1 — mt2c00830_si_001.pdf [file mt2c00830_si_001.pdf]

Supporting information

Single-Particle Functionality Imaging of Antibody-Conjugated Nanoparticles in Complex Media

Laura Woythe[1], Marrit M.E. Tholen[1], Bas J.H.M. Rosier[1], Lorenzo Albertazzi[1,2*]

[1]Department of Biomedical Engineering, Institute for Complex Molecular systems (ICMS), Eindhoven University of Technology;

[2]Institute of Bioengineering of Catalonia (IBEC), the Barcelona Institute of Science and Technology (BIST)

*** l.albertazzi@tue.nl**

Supplementary Figures

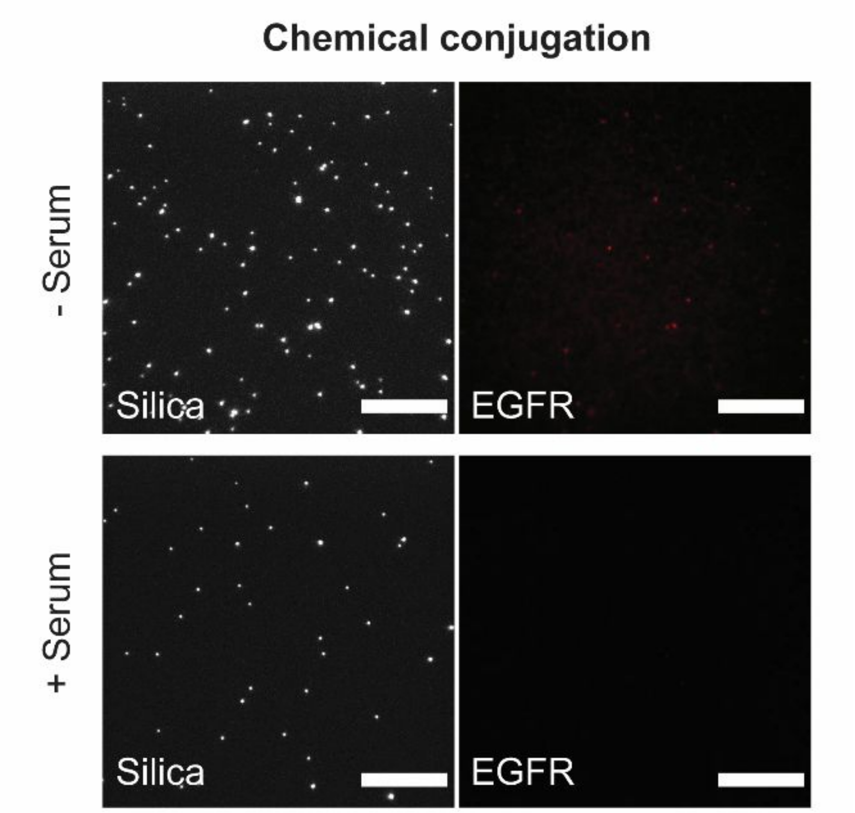

Supplementary Figure S1. TIRF images of EGFR-AF647 probe binding to silica NPs chemically functionalized with human IgG control antibody in the presence and absence of serum. Silica NP fluorescence is displayed in greyscale and EGFR-AF647 in red. Scale bar 20 μm.

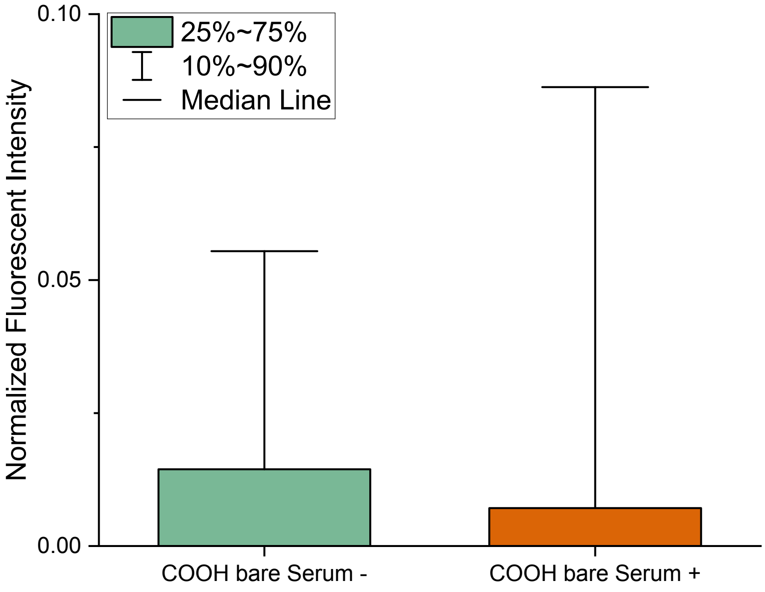

Supplementary Figure S2. Control for the evaluation of NP functionality using the bare particles that were used for synthesis of chemically conjugated particles, represented in box plots, with and without serum incubation. Box represents 25 to 75 percentile and whiskers 10 to 90 percentile. Small square indicated mean normalized fluorescent intensity. A minimum of 587 particles was analyzed.

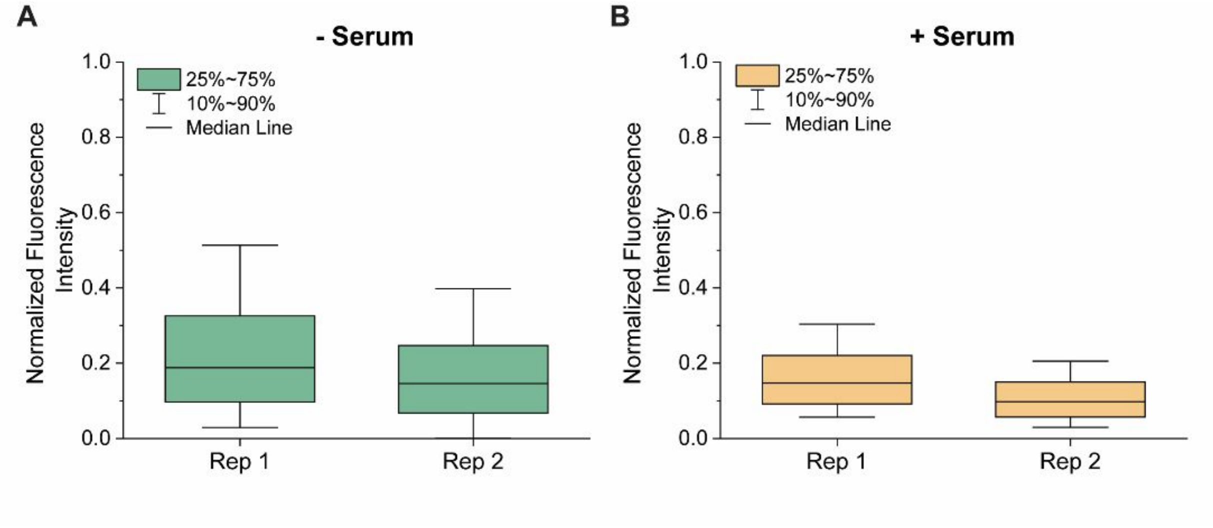

Supplementary Figure S3. Reproducibility of TIRF imaging of silica-cetuximab NPs incubated with EGFR-AF647 in A) the absence and B) the presence of serum. Rep 1 corresponds to data shown in Figure 2 and Rep 2 to data displayed in Figure 3B, i). In both panels, cetuximab was conjugated to NPs via EDC chemical conjugation at 1000 cetuximab/ NP.

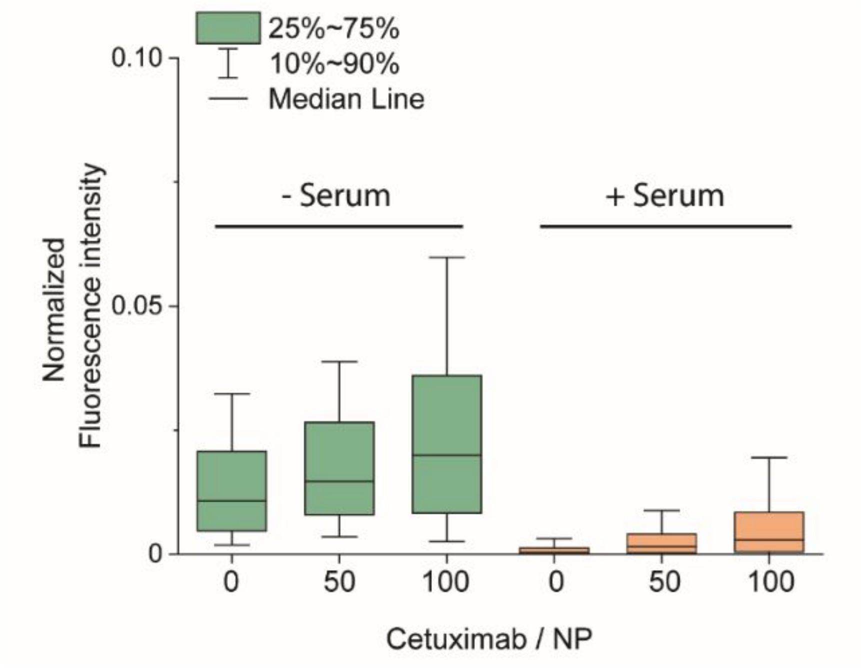

Supplementary Figure S4. Zoom-in view of Y-axis in Figure 2, representing the normalized fluorescence intensity box plots for NP functionality at low cetuximab functionalization without and with serum.

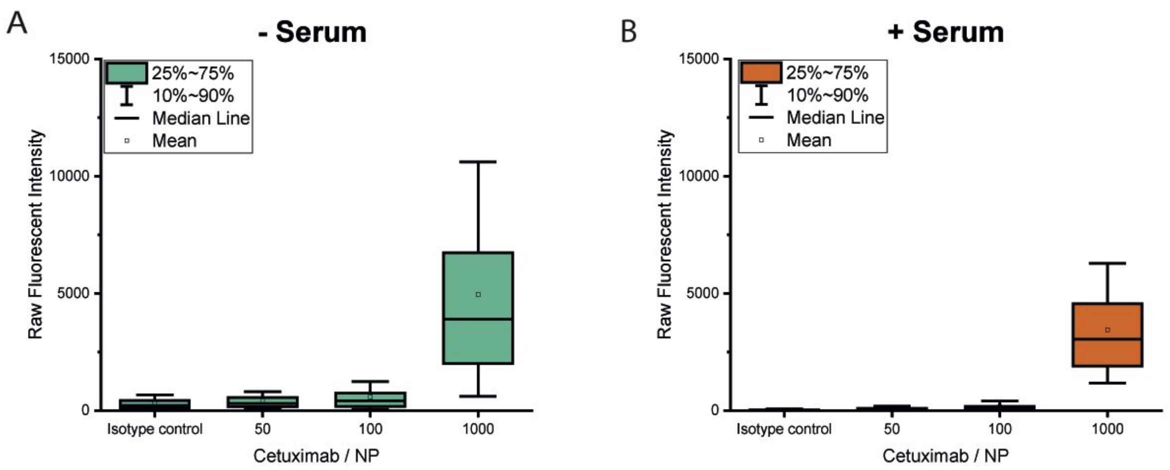

Supplementary Figure S5. Raw data plots of the results shown in Figure 2, validating the normalization. NP functionality in A) the absence or B) in the presence of serum. Box represents 25 to 75 percentile and whiskers 10 to 90 percentile.

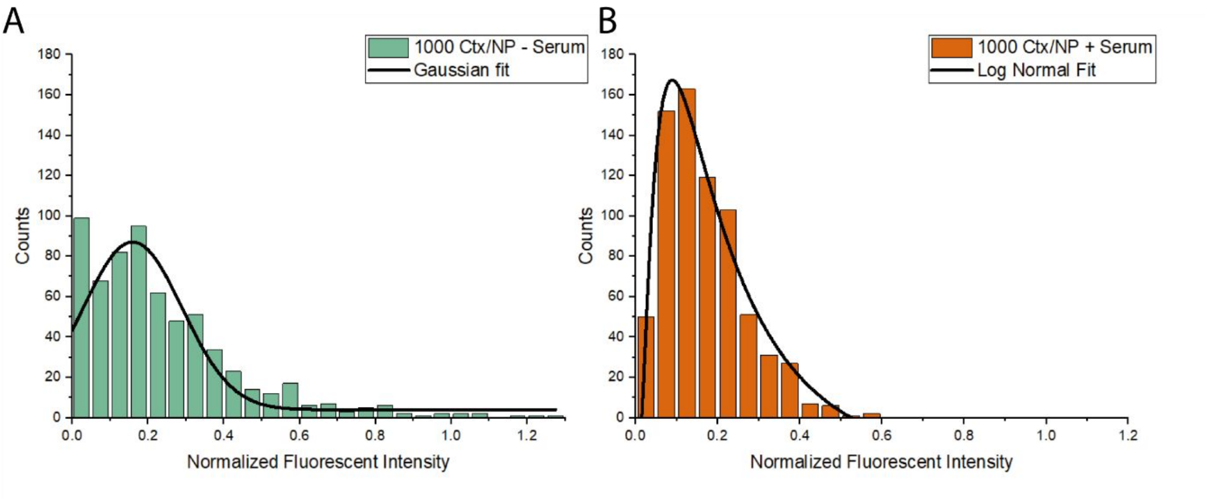

Supplementary Figure S6. Curve fits for the data represented in Figure 2. A) 1000 Cetuximab / NP without Serum, fitted with a Gaussian curve, R^2=0.8, B) 1000 Cetuximab / NP with serum, fitted with a Log Normal distribution, R^2=0.97.

Table S1: t-test performed on the results of Figure 2, p-values are recorded.

**t-test**	**p-value**
Isotype – 50 (-Serum)	$2.46*10^{-6}$
Isotype – 100 (-Serum)	$7.26*10^{-16}$
Isotype – 1000 (-Serum)	$1.66*10^{-167}$
50 – 100 (-Serum)	$1.04*10^{-5}$
50 – 1000 (-Serum)	$1.54*10^{-216}$
100 – 1000 (- Serum)	$1.02*10^{-281}$
Isotype – 50 (+Serum)	$8.36*10^{-33}$
Isotype – 100 (+Serum)	$3.98*10^{-44}$
Isotype – 1000 (+Serum)	0
50 – 100 (+Serum)	$2.02*10^{-8}$
50 – 1000 (+Serum)	0
100 – 1000 (+Serum)	0
Isotype (- Serum) – Isotype (+Serum)	$1.31*10^{-111}$
50 (- Serum) – Isotype (+Serum)	$5.22*10^{-167}$
100 (- Serum) – 100 (+ Serum)	$1.15*10^{-88}$
1000 (- Serum) – 1000 (+ Serum)	0.0264

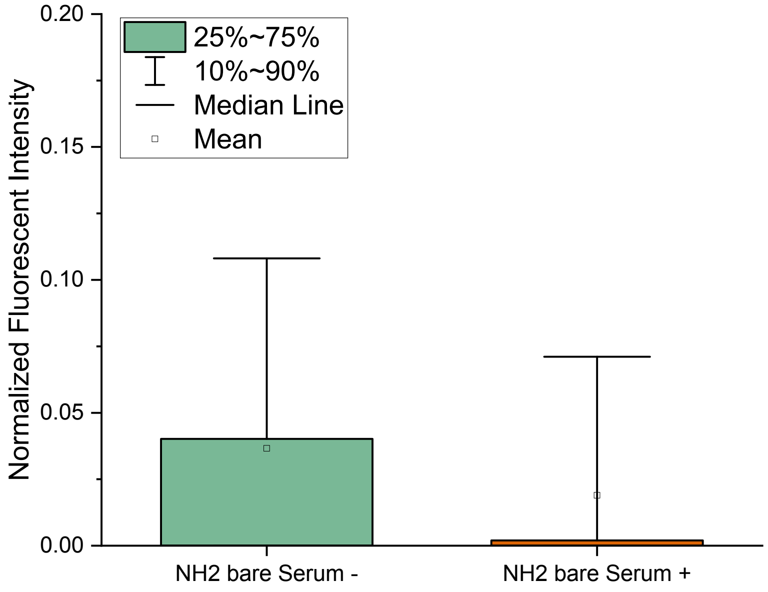

Supplementary Figure S7. Negative control for the evaluation of NP functionality using the bare particles that were used for synthesis of physical adsorbed and the protein G particles, represented in box plots, with and without serum incubation. Box represents 25 to 75 percentile and whiskers 10 to 90 percentile. Small square indicated mean normalized fluorescent intensity. A minimum of 583 particles was analyzed.

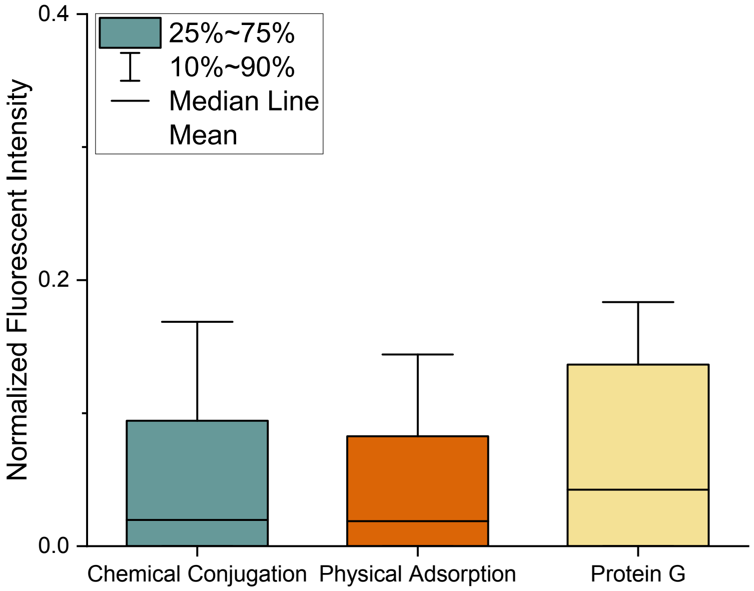

Supplementary Figure S8. Protein corona formation on antibody conjugated particles, performed with fluorescently labeled FBS. Box represents 25 to 75 percentile and whiskers 10-90 percent percentile.

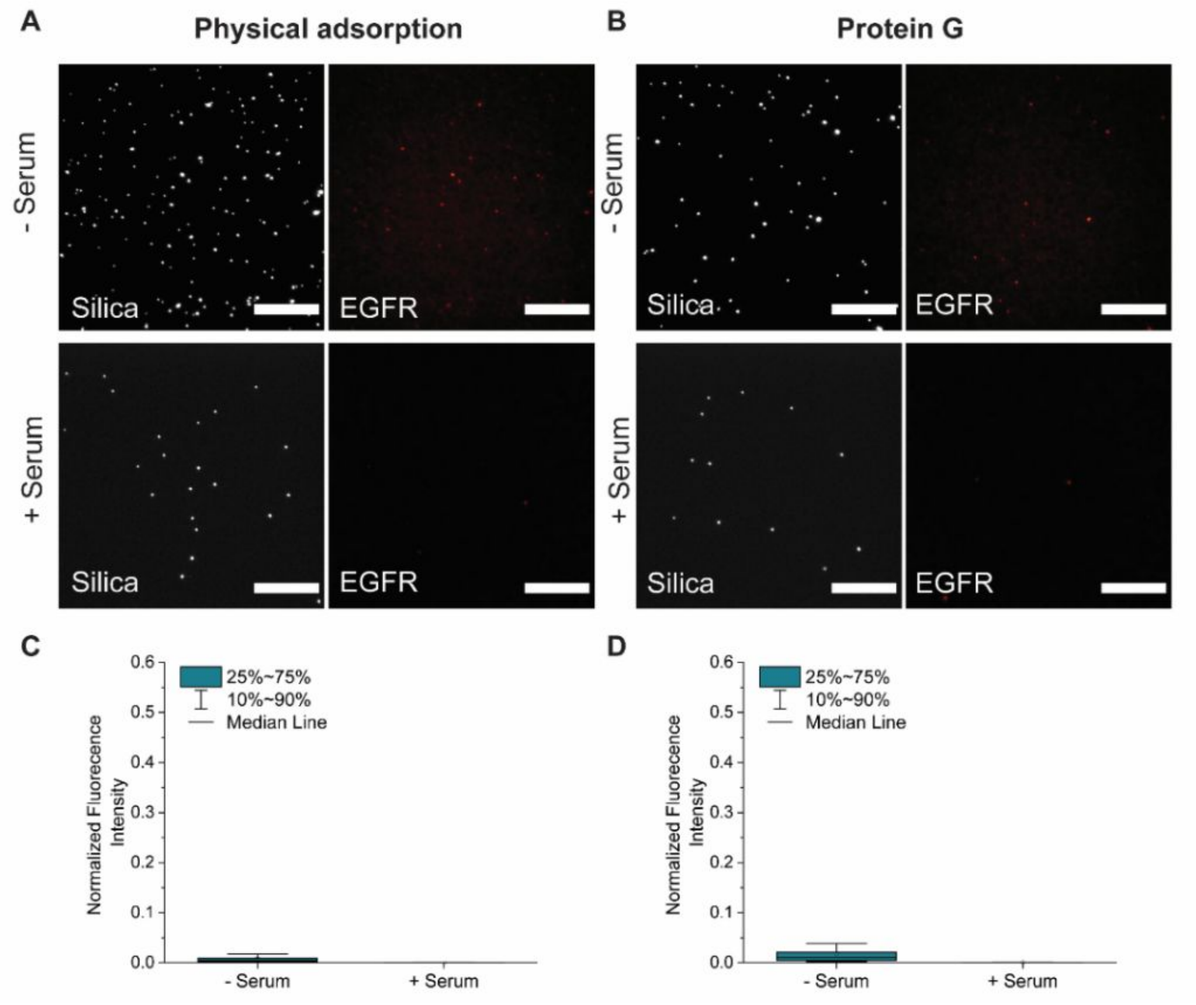

Supplementary Figure S9. EGFR-AF647 probe binding to control silica NPs conjugated with human IgG control antibody via A) physical adsorption or B) Protein G-mediated adsorption in the presence and absence of serum. Silica NP fluorescence is displayed in greyscale and EGFR-AF647 in red. Scale bar 20 μm. C) and D) represent box plots quantifying the normalized fluorescence intensity from conditions A) and B), respectively.

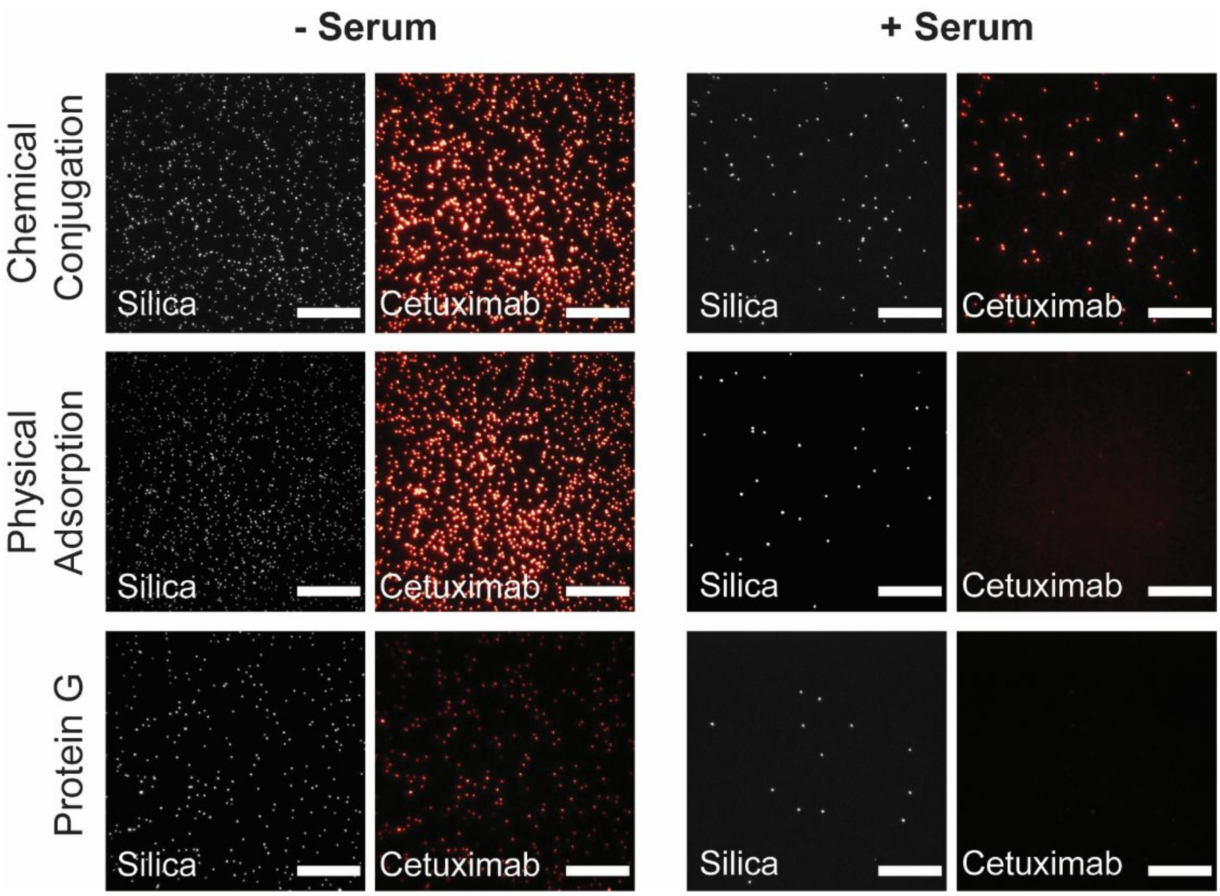

Supplementary Figure S10. Conjugation of cetuximab-AF647 to silica NPs via chemical conjugation, physical adsorption and protein G before (-serum) and after (+serum) exposure to 100% serum. In the presence of serum, NP binding to the glass surface is reduced due to surface blocking of serum components. NP position (Silica) is shown in grey and cetuximab-AF647 signal in red. Scale bar 20 µm.

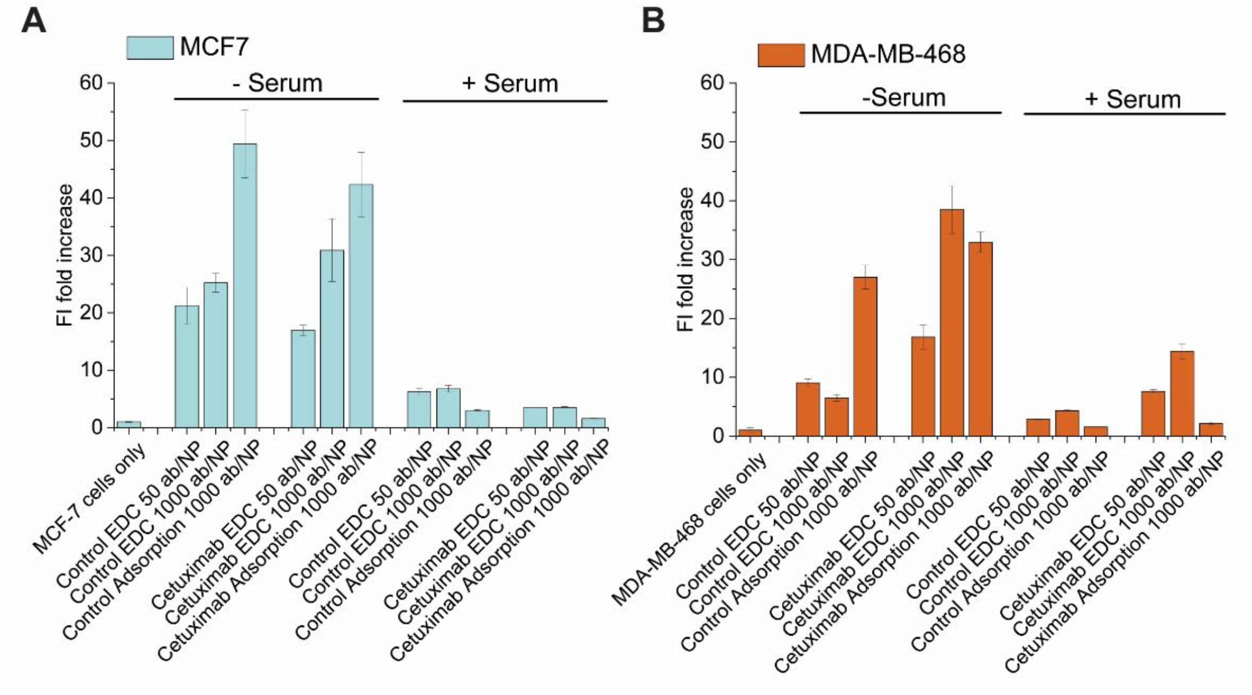

Supplementary Figure S11. Fluorescence intensity (FI) mean fold increase of NP-exposed cells respect to cells with no NPs as measured by flow cytometry A) in MCF-7 and B) in MDA-MB-468 cells without and with serum pre-incubation. Control conditions represent NPs conjugated with a human isotype control IgG antibody.

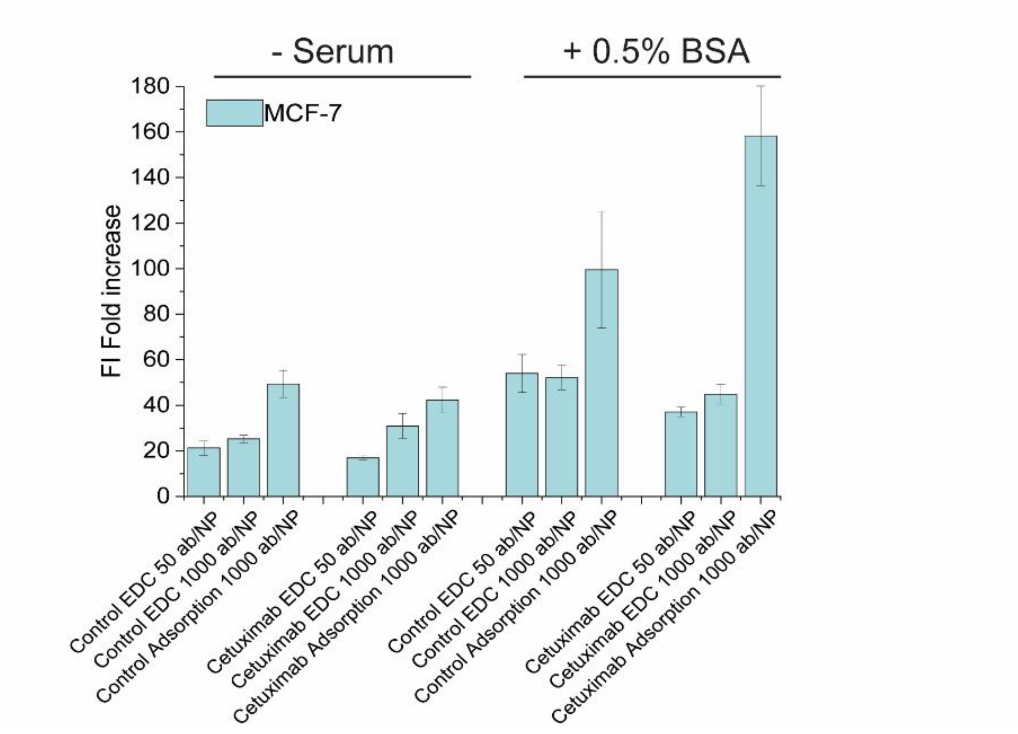

Supplementary Figure S12. Fluorescence intensity (FI) mean fold increase of NP-exposed cells respect to cells with no NPs as measured by flow cytometry in MCF-7 without serum pre-incubation and with 0.5% BSA blocking solution. Control conditions represent NPs conjugated with a human isotype control IgG antibody.

atgggctggtcccatccgcagttcgagaaaggtaccatgacatttaaactgataatcaac
M G W S H P Q F E K G T M T F K L I I N
ggcaaaaccttaaaaggggagatcacaattgaggcagtcgatgcctaggaagccgagaaa
G K T L K G E I T I E A V D A - E A E K
atctttaaacaatatgctaatgattatggtattgacggagaatggacgtatgacgatgcg
I F K Q Y A N D Y G I D G E W T Y D D A
acaaaaactttcaccgtaactgaggaattcactagtggtggaagtggggacgatcatcat
T K T F T V T E E F T S G G S G D D H H
catcatcatcattgc**tga**
H H H H C -

Supplementary Figure S13. DNA and amino acid sequence of pG. Areas of interest are indicated as follows: Strep-tag (magenta), protein G (yellow), amber stop codon (bold), His-tag (grey), cysteine site (green).

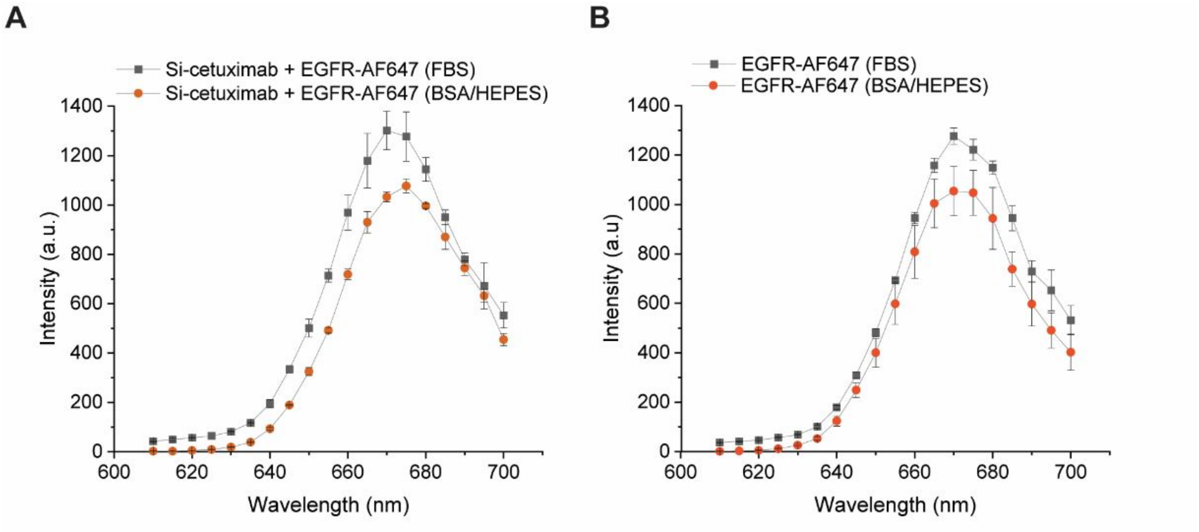

Supplementary Figure S14. Fluorescence intensity of EGFR-AF647 probe in full serum (FBS) (grey) or in HEPES/BSA buffer (orange). A) Fluorescence intensity of EGFR-AF647 probe attached to silica-cetuximab NPs. B) Fluorescence intensity of EGFR-AF647 probe free in solution.
